# Effect of Gut Dysbiosis on Onset of GI Cancers

**DOI:** 10.3390/cancers17010090

**Published:** 2024-12-30

**Authors:** Seema Kumari, Mundla Srilatha, Ganji Purnachandra Nagaraju

**Affiliations:** 1 Cancer Biology Laboratory, Department of Biochemistry and Bioinformatics, GIS, GITAM (Deemed to Be University), Visakhapatnam 530045, Andhra Pradesh, India; 2 Department of Biotechnology, Sri Venkateswara University, Tirupati 517502, Andhra Pradesh, India; 3 Division of Hematology and Oncology, School of Medicine, University of Alabama at Birmingham, Birmingham, AL 35233, USA; purnachandraganji@uabmc.edu

**Keywords:** GI cancer, dysbiosis, gut microbiota, FMT, CRCbiome

## Abstract

Dysbiosis in the gut microbiota plays a significant role in GI cancer development by influencing immune function and disrupting metabolic functions. Dysbiosis can drive carcinogenesis through pathways like immune dysregulation and the release of carcinogenic metabolites, and altered metabolism, genetic instability, and pro-inflammatory signalling, contributing to GI cancer initiation and progression. *Helicobacter pylori* infection and genotoxins released from dysbiosis, lifestyle and dietary habits are other factors that contribute to GI cancer development. Emerging diagnostic and therapeutic approaches show promise in colorectal cancer treatment, including the multitarget faecal immunochemical test (mtFIT), standard FIT, and faecal microbiota transplantation (FMT) combined with PD-1 inhibitors. We used search engine databases like PubMed, Scopus, and Web of Science. This review discusses the role of dysbiosis in GI cancer onset and explores strategies such as FMT, probiotics, and prebiotics to enhance the immune response and improve cancer therapy outcomes.

## Simple Summary

Cancers of the gastrointestinal (GI) tract are most aggressive, with poor prognosis and survival rates. The main factors that contribute to cancer development are genetic modifications, *Helicobacter* infection, gut dysbiosis, inflammation, and altered drug metabolism. Microbial metabolites like SCFA and secondary bile acids have dual roles, and a disruption in microbial balance may contribute to cancer progression. Understanding microbiota–tumour interactions is crucial in cancer therapies. Chronic inflammation promotes cancer progression and resistance to therapy, while acute inflammation activates antitumour activity. Significant pathways such as NF-κB, JAK-STAT, cGAS/STING, cytokines, chemokines and microbial metabolites control inflammation. Modern strategies in cancer treatment are use of recombinant cytokines, small-molecule inhibitors, and dendritic cell vaccines which target the signal pathways. Probiotics play a pivotal role in chemotherapy-induced gastroenteritis and in restoring gut microbiota balance. Moreover, reducing digestive disorders can improve the diversity of gut flora, improve thyroid hormone, and have antitumour activity by regulating the TLR4/MyD88/NF-κB pathway. Developing evidence emphasises on the gut microbiota’s diagnostic and therapeutic potential in screening for colorectal cancer, treatment outcomes, and prevention strategies.

## 1. Introduction

Gastrointestinal (GI) cancers are the most prevalent form of cancer globally and contribute to 8% of overall deaths [1]. GI cancers have a dismal prognosis of 10–20% and a 5-year survival rate, mostly because they are often detected at an advanced stage and are inherently aggressive [2]. Distinct genetic maps and signal transduction are involved in the development and progression of GI cancers. Likewise, dietary habits and infection with *Helicobacter pylori* are environmental factors that substantially impact the growth of GI cancers. These factors interact with genetic and epigenetic changes that affect carcinogenic pathways, such as mutations, altered gene methylation, and genetic instability, causing chromosomal or microsatellite instability [3,4].

A plethora of microorganisms such as commensal bacteria, viruses, fungi, and archaea make up the gut microbiota, which is essential to the maintenance of a healthy environment and also impacts the occurrence of ailments, including GI cancer [5]. Microbial dysbiosis influences carcinogenesis by affecting immune function and environmental factors. The gut microbiota plays a crucial role in immune system development, with the involvement of intestinal epithelial cells (IECs) detecting microbial growth and triggering immune responses. Dysbiosis also disrupts immune homeostasis, while gut bacteria can directly impact systemic cancer immunity [6]. In turn, cancer cells release metabolites that alter gut microbiota, influencing the tumour microenvironment and promoting immune suppression [7]. Manipulation in the environmental milieu can impact immune response, host metabolism, and the formation of tumours. Thus, cancer therapies involve targeting the altered metabolism, which is triggered by mutations and metabolic reprogramming [8]. Obesity and hypertension are additional ailments that increase the risk of cancer. Cancer can result from dysbiosis or microbial imbalance; for instance, colorectal cancer is frequently associated with infections like *Escherichia coli* [9]. Comprehending the ways in which gut bacteria communicate with cancerous cells and modulate immune responses is crucial for improving cancer treatment outcomes, as it impacts tumour development and the efficiency of cancer medicines.

The colon is a habitat for intricate groups of gut microbiomes, including bacteria, viruses, fungi, and archaea. Dysbiosis has been linked to several illnesses, including cancer, which highlights the significance of maintaining a diverse and balanced microbiota for overall health. Therefore, the relationship between microbes and tumours has gained attention in the area of anticancer treatment, carcinogenesis, and cancer prevention [10]. There are several factors involved in the cancer susceptibility caused by microbial dysbiosis; since the microbiota induces inflammation and immunologic dysregulation, generates genetic instability, and alters the pharmacodynamics of anticancer medications, the microbiota and its metabolites are intimately linked to the development of cancer [11]. Chronic inflammation and the release of cytokines are associated with various cancers, including myeloproliferative neoplasms [12]. Moreover, chronic inflammation causes cancer development by influencing TME, wherein pro-inflammatory cytokines like IL-6 and MIF stimulate oncogenic pathways and promote immune escape. Cancer cells seize regulatory immune cells, such as Tregs and Bregs, to induce local and systemic immunosuppression. Even though the developments in chemotherapy, radiotherapy, and immune checkpoint therapy have improved cancer treatment, the efficacy remains limited due to drug resistance and the immunosuppressive TME [13].

Due to the possibility that gut microbiota may influence cancer through pathways including inflammation, immunological control, and metabolic processes, further research on the potential connections between GI malignancies and the gut microbiota is hopeful, since microbial dysbiosis plays a major role in carcinogenicity [14]. Prolonged inflammation has the potential to accelerate tumour growth, infiltration, and dissemination, leading to DNA damage, aberrant methylation, and the involvement of pro-inflammatory cytokines such as TNF-α, IL-1, IL-6, and IL-10. These cytokines modify the transition of epithelial cells by increasing oxidative stress, sensitising STAT3, and preventing apoptosis. Signal pathways such as NF-κB, Wnt, and MAPK kinase are stimulated in addition to inflammatory substances, which deactivate tumour-suppressor genes and stimulate oncogenes [15].

Tumour formation is significantly influenced by innate and adaptive immunity induced by dysbiosis of the gut microbiota. Carcinogenesis is aided by the adaptive immunological activity of T regulatory (Treg) cells, Th helper (Th) cells, and secretory immunoglobulin A (IgA) [16]. Microbial metabolites such as short-chain fatty acids (SCFAs), lipoteichoic acid (LTA), and secondary bile acids play dual roles in cancer development. Cell division, DNA damage, and cellular senescence are all activated by secondary bile acids’ activation of GPBAR1, which is triggered by LTA’s binding to CD14 or TLR2. In contrast, SCFAs help immunoregulation through T regulatory (Treg) cells, which in turn has anti-inflammatory and anti-carcinogenic properties [17]. A previous review discussed gut microbiota and various biomarkers showing the cancer progression and combination approach of microbiota and metabolites in cancer diagnosis [9]. This review will discuss the that contributes to GI cancers like gut dysbiosis, inflammation, and altered drug metabolism. Also, the role of microbial metabolites like SCFAs and bile acids is discussed in GI cancer progression. Key inflammatory pathways, including NF-κB, JAK-STAT, and cGAS/STING, targeted by modern therapies like cytokines, inhibitors, and vaccines, are also discussed in the review. The role of probiotics in restoring gut balance and mitigating chemotherapy effects is also discussed.

## 2. Gut Microbiome and Gastrointestinal Health

The major constituents of gut microbiota are Firmicutes, Bacteroidetes, Actinobacteria, Fusobacteria, Proteobacteria, Verrucomicrobia, and Cyanobacteria, with Bacteroidetes and Firmicutes as major taxa. Bacteroidetes include the genera *Bacteroides* and *Prevotella*, while Firmicutes contain *Clostridium*, *Eubacterium*, and *Ruminococcus* genera. *Methanobrevibacter smithii* and *Haloferax alexandrinus* are two distinct kinds of archaea. The human microbiome is composed of three enterotypes: *Ruminococcus*, *Prevotella*, and *Bacteroides* [18]. Dietary and external factors affect these enterotypes. Diverse acid-resistant bacteria, including *Helicobacter pylori*, *Lactobacillus*, *Neisseria*, and *Streptococcus*, have been identified in the stomach. Since bile acids lead the duodenum to possess low bacterial density, the jejunum has a wider range of bacteria, and the ileum has an increased number of anaerobic microbes [19]. Firmicutes and Viridites predominate in the large intestine and are important for fermentation and water absorption. Notable genera in this group include *Bacteroides*, *Bifidobacterium*, and *Clostridium*. This equilibrium can be upset by medication, food, or disease, and some microbial signatures are linked to ageing in a healthful manner. Core microbial species like *Faecalibacterium prausnitzii* and *Bacteroides uniformis* are dynamic in gut health, exhibiting functional redundancy across individuals [20]. Microecological factors also impact the microbial composition. The gut microbiota operates in metabolic, protective, structural, and neurological landscapes fermenting dietary fibres, producing SCFAs, influencing the gut–brain axis, and regulating the immune system. The protective function involves the mucin, antimicrobial peptides, and secretory IgA, which maintain the intestinal barrier and prevent pathogen adherence [21]. The immune response discriminates between commensals and pathogens through pattern-recognition receptors and dendritic cells and maintains immune balance and tolerance. Therapeutic interventions like probiotics, prebiotics, and faecal microbiota transplantation (FMT) offer an avenue for modulating gut microbiota and improving health outcomes. Dysbiosis is linked to diseases like IBD, obesity, diabetes, and cancer, including gastric cancer, highlighting the microbiota’s role in health and disease [6,10]. Table 1 provides the list of bacteria undergo dysbiosis in GI cancers.

## 3. Gut Microbiota’s Influence on Cancer Progression

### 3.1. Inflammation

**Figure 1:**
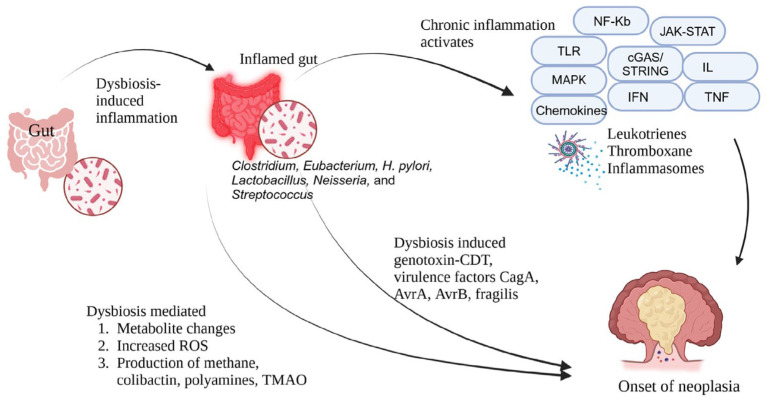
Chronic inflammation in onset of neoplasia. Inflammation significantly impacts tumour development, often by activating signal pathways like NF-kB, JAK-STAT, TLR, cGAS/STING, and MAPK alongside cytokines, chemokines, leukotrienes, thromboxane and inflammatory metabolites, which regulate inflammation. The dysbiosis-induced metabolic changes in short-chain fatty acids, bile acids, ROS, methane, colibactin, polyamines, lithocholic acid, butyrate, and trimethylamine N-oxide (TMAO) regulate cancer onset. Dysbiosis-induced genotoxin-CDT and virulence factors CagA, AvrA, AvrB, and fragilis induce cancer onset.

Inflammation plays a crucial role in regulating the neoplasia and its response to therapy by exerting tumour-promoting or tumour-suppressing effects that can influence therapeutic outcomes. Chronic inflammation tends to support tumour progression and resistance to treatment, while acute inflammatory reactions can stimulate the activation of DCs and antigen presentation, thereby triggering antitumour immune responses [22]. Various signal transduction pathways, including nuclear factor kappa B (NF-kB), Janus kinase/signal transducers and activators of transcription (JAK-STAT), Toll-like receptor (TLR) pathways, cGAS/STING, and mitogen-activated protein kinase (MAPK), along with inflammatory factors such as cytokines (e.g., interleukin (IL), interferon (IFN), tumour necrosis factor (TNF)-α), chemokines (e.g., C-C motif chemokine ligands (CCLs) and C-X-C motif chemokine ligands (CXCLs)), growth factors (e.g., vascular endothelial growth factor (VEGF), transforming growth factor (TGF)-β), and inflammasomes, as well as inflammatory metabolites like prostaglandins, leukotrienes, thromboxane, and specialised pro-resolving mediators (SPMs), have been identified as critical regulators of inflammation initiation and resolution [23]. Modern cancer therapeutic strategies now include local irradiation, recombinant cytokines, neutralising antibodies, small-molecule inhibitors, DC vaccines, oncolytic viruses, TLR agonists, and SPM to modulate inflammation specifically [24]. Many of these approaches are undergoing clinical trials. This argument delves into the intricate mechanisms behind inflammation initiation and resolution and the interplay between tumour development and inflammatory processes (Figure 1). Furthermore, potential targets for manipulating inflammation in cancer treatment are highlighted [15].

### 3.2. Genotoxicity

Bacterial genotoxins such as cytolethal distending toxin (CDT), typhoid toxin, and colibactin (Figure 1) induce DNA damage similar to mammalian DNase I activity, triggering the DNA damage response (DDR) and potentially leading to cell senescence, apoptosis, or genomic instability, thus facilitating tumour initiation and progression [25]. CDT, for instance, halts the eukaryotic cell cycle at the G2/M transition, categorised as cyclomodulins, and is delivered to the nucleus by CdtA and CdtC, activating DDR, promoting genomic instability, and disrupting the cell cycle. Studies on CDT prevalence across various species reveal its role in tissue colonization and evasion of host defences by disrupting epithelial barriers and impairing immunity. The active subunit CdtB shares structural similarity with DNase I and induces DNA double-strand breaks (DSBs), activating DDR through ATM. CdtA and CdtC facilitate holotoxin binding to the cell membrane, potentially via lipid rafts, which facilitates CdtB translocation to the nucleus to initiate DDR, cell cycle arrest, and potential carcinogenesis, emphasising its involvement in tumour progression [26,27].

Infectious agents may contribute to cancer development, with bacterial toxins disrupting the cellular signals involved in oncogenesis, such as cell proliferation, cell cycle progression, and DNA repair. Notable examples include *Helicobacter pylori*, *Salmonella* species, and *Enterotoxigenic Bacteroides fragilis*, which secrete virulence factors like CagA, AvrA and -B, and fragilis toxin, respectively, promoting carcinogenesis [28]. *Helicobacter hepaticus* infection leads to chronic hepatitis and dysplasia, with CDT upregulating pro-inflammatory mediators and anti-apoptotic proteins, ultimately fostering tumour formation. Mechanistically, CDT-induced DDR initiates cell cycle arrest, but persistent DNA damage can drive malignant transformation characterised by disrupted genetic stability, increased mutation risk, and micronuclei formation, further fuelling inflammation through the cGAS-STING pathway. The master regulator ATM activates survival pathways like p38 MAPK and integrin β1 and interacts with the cytoskeleton via RhoA signalling, facilitating tumour invasion and metastasis [29,30]. Recent evidence suggests a role for CDT in metastasis, with gut bacteria disseminating to the liver, recruiting immune cells, and establishing a premetastatic niche. Infection with CDT-producing bacteria can compromise the intestinal barrier, enabling surviving tumour cells to migrate to the liver with the bacteria, promoting metastasis in the inflammatory microenvironment [31,32]. These findings underscore the imperative need to further elucidate the mechanisms underlying bacterial toxin-induced cancer development.

### 3.3. Metabolism and Carcinogenesis

The synthesis of small-molecule metabolites by the microbiota is essential for developing and inhibiting cancer. SCFAs, bile acids (BAs), reactive oxygen species (ROS), and methane are illustrations of primary metabolites that play a role in growth and have the potential to affect the course of cancer [33]. Since colibactin causes DNA double-strand breaks, secondary metabolites like thiopeptides and colibactin have more specific roles and may contribute to cancer. Produced by bacterial fermentation, SCFAs affect immunological responses and, depending on the situation, can either promote or inhibit cancer [34]. Bile acids are linked to DNA damage and oncogenic alterations, especially secondary bile acids like deoxycholic acid. Furthermore, gut bacterial-produced compounds such as methylglyoxal (MGO) and polyamines affect immune modulation, tumour resistance, and the survival of cancer cells, underscoring the complex connections between the microbiome and the tumour microenvironment [35]. Understanding these microbial metabolites can help identify novel treatment targets and provide novel insights into the progression of cancer.

**Figure 2:**
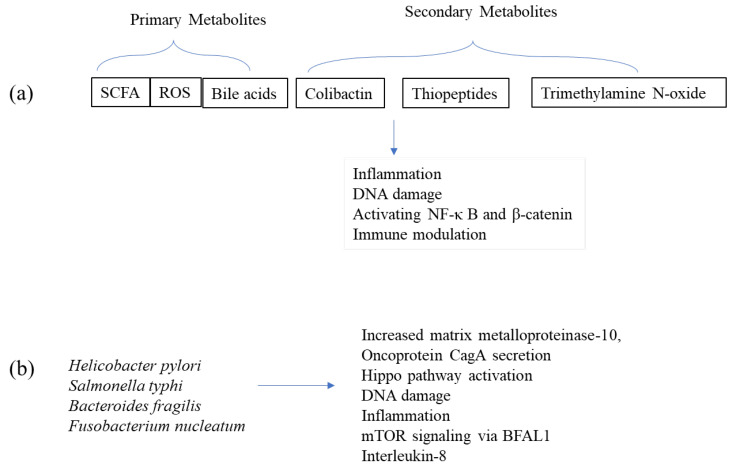
Metabolites and microbiota in cancer progression—(**a**) Primary and secondary metabolites play an important role in inflammation, DNA damage, activating NF-κ B and β-catenin, and immune modulation. (**b**) Altered colon microbiota are linked to cancers. For example, *Helicobacter pylori* contributes to gastric cancer via increased matrix metalloproteinase-10, oncoprotein CagA secretion, and Hippo pathway activation. *Salmonella typhi* causes DNA damage and inflammation. *Bacteroides fragilis* activates mTOR signalling via BFAL1, and *Fusobacterium nucleatum* enhances interleukin-8 secretion, supporting tumour proliferation and migration.

The development of colorectal cancer is complicatedly linked to bile acid metabolism, which is impacted by the gut microbiota. The liver releases primary beta-amylases (BAs) such as cholic acid (CA) and chenodeoxycholic acid (CDCA), which are converted by the gut bacteria into secondary BAs, such as deoxycholic acid (DCA) and lithocholic acid (LCA); these are associated with promoting colorectal cancer by triggering inflammation, disrupting DNA, and activating pathways including NF-κB and β-catenin [36]. These secondary BA products are primarily generated by bacteria, such as *Clostridium* species. LCA, produced by Clostridium, aids in immunological evasion and oxidative stress in colorectal cancer [36]. Bile salt hydrolases and other gut bacteria, including *Bacteroides fragilis* and *Lactobacillus*, are important players in the metabolism of BAs [37]. Moreover, butyrate, which is produced by *Roseburia intestinalis*, *Faecalibacterium prausnitzii*, and *Butyrivibrio crossotus*, is a form of a short-chain fatty acid (SCFA) that acts as a protective metabolite by maintaining the integrity of the gut barrier, decreasing inflammation, and controlling immune responses, all of which hinder the progression of colorectal cancer [38]. The relationship between gut bacteria and these compounds emphasises how important the microbiome is in regulating cancer risk (Figure 2).

SCFAs like acetate and propionate, which are generated during the fermentation of fibre, have anti-colorectal cancer effects. Acetate, which bacteria like *F. prausnitzii*, *Roseburia* spp., and *Coprococcus* sp. convert to butyrate, inhibits the progression of colorectal cancer by increasing the syntrophy of the gut microbiota [39]. Propionate, an HDAC inhibitor, induces precancerous colonic cells to undergo apoptosis; nevertheless, it is less effective than butyrate and is less common in colorectal cancer. On the contrary, formate—an oncometabolite generated from the gut—is released by *Fusobacterium nucleatum*, and promotes the invasion of colorectal cancer by stimulating the Aryl hydrocarbon receptor and enhancing cancer stemness [39]. Trimethylamine N-oxide (TMAO), another microbial metabolite, produced from dietary l-carnitine and phosphatidylcholine by Enterobacteriaceae, has been linked to a higher risk of CRC. Research on the relationship between elevated TMAO levels and poor mortality in CRC patients has produced mixed findings. Microbial metabolites influence the growth of tumours, inflammation, and immunity [40]. Also, modifications to amino acid metabolism, especially that of tryptophan, serine, and branched-chain amino acids, are linked to the development of CRC. Finally, the gut microbiota’s degradation of metabolites from dietary phytochemicals has anti-inflammatory and antibacterial properties that affect gut health and the development of CRC. Crucial virulence factors, such as FadA (Fusobacterium adhesin A) and Fap2, made by *Fusobacterium nucleatum* and *C. perfringens*, respectively, interact with immunological and epithelial cells to promote CRC, highlighting the complex involvement of the gut microbiota in CRC formation [41,42].

### 3.4. Evidence Linking Colon Microbiota to Cancer

GI cancers include cancers of the mouth, oesophagus, stomach, liver, pancreas, colon, and rectum. As mentioned earlier, these cancers are often linked to risk factors such as chronic inflammation, infections (e.g., *Helicobacter pylori*), diet, alcohol consumption, smoking, and genetic mutations. Early-stage digestive tract cancers may not show symptoms, making early detection challenging. Colorectal cancer is one of the most common types, with risk factors including a poor diet, family history, and gut microbiota imbalances. Chronic conditions like inflammatory bowel disease (IBD) also increase cancer risk [43]. Numerous studies have linked alterations in the colon microbiota to various cancers, including colorectal and gastric cancers. For instance, a higher abundance of *Helicobacter pylori* is a well-established risk factor for gastric cancer as it increases the level of matrix metalloproteinase-10 in the gastric mucosa, promoting its colonization, and secretes the oncoprotein cytotoxin-associated gene A, which activates the Hippo pathway and leads to chronic inflammation [4,44]. *Salmonella typhi* is closely associated with biliary cancer development by secreting multiple virulence factors that cause DNA damage and induce inflammation. Gut microbes also disrupt the metabolic balance of bile acids, converting primary bile acids into secondary bile acids, which impair natural killer T cells’ immune ability, promoting tumour growth [45]. In colon cancer, enterotoxigenic *Bacteroides fragilis* activates mTOR signalling via the long non-coding RNA BFAL1, accelerating tumour growth, while *Fusobacterium nucleatum* promotes the secretion of interleukin-8 and C-X-C motif chemokine receptor 1, supporting the proliferation and migration of HCT116 cells [46]. Thus, the microbiome is increasingly crucial in tumour progression and cancer treatment, as shown in Figure 2.

The gut microbiota is largely shaped by diet, and, in turn, affects the molecular activities of the colonic mucosa. An important element in the pathophysiology of colorectal cancer is an unbalanced microbiome, which is linked to compromised immune responses and carcinogenic consequences. SCFAs, produced by the microbiota from dietary fibres, have anti-inflammatory and antiproliferative properties that promote colon health [47]. Conversely, diets deficient in fibre and phytochemicals, along with a greater consumption of red meat, increase the risk of colorectal cancer [48]. By controlling carcinogenic pathways, a balanced gut microbiota can slow down the growth of new cells. By limiting the growth of cells, cancerous networks are frequently regulated by a harmonious gut microbiota [49]. Good lifestyle choices can prevent the proliferation of hazardous bacteria like *Fusobacterium nucleatum*, which may negatively impact colon health. Synbiotics are a combination of prebiotics and probiotics that improve immune function, lower toxic metabolites, and reduce oxidative stress. This emphasises that promoting healthy gut microbiota through a plant-based diet might help prevent colorectal cancer [50]. In CRC, the gut–liver axis is associated with liver metastasis, whereas neutrophil extracellular traps (NETs) are connected to tumour metastasis. Tumour growth-promoting inflammation in the liver can result from bacterial translocation triggered by abnormalities in the gut barrier [51]. The gut’s xenobiotic metabolism is controlled by the aryl hydrocarbon receptor (AhR) and pregnane X receptor (PXR), which are also connected to inflammation and equilibrium in the gut. They could avert CRC spurred on by inflammation, but they also alter colon cell behaviour, which may accelerate the development of the disease. Since their malfunction can result in dysbiosis and chronic inflammation, type 3 innate lymphoid cells (ILC3s) are connected to colorectal cancer and help maintain the gut microbiota equilibrium [52]. By promoting antitumour immunity, ILC3s also help immune checkpoint inhibitor (ICI) medications work well. New research indicates that the gut microbiota influences how the body reacts to immune checkpoint inhibitors (ICIs) via interacting with immune cells or by using processes unique to particular antigens [53]. These findings underline the complicated involvement of the microbiota in CRC and treatment success.

## 4. Clinical Evidence

The influence of probiotics in lowering gastroenteritis and reviving an equilibrium of the gut microbiota was studied in patients undergoing chemotherapy for colorectal cancer. The first group of one hundred CRC patients received probiotics, whereas the other group received a placebo. The probiotic group noticed a substantial reduction in digestive disorders, such as diarrhoea (*p* < 0.01). Probiotics increased the number of *Bifidobacterium* and other beneficial bacteria and encouraged the synthesis of SCFAs, helping to restore the variety and composition of the gut flora that chemotherapy had damaged [54]. The second research investigated the impact of the herbal compound Xiao-Chai-Hu-Tang (XCHT) on individuals suffering from depression during cancer. XCHT alleviated depression symptoms, decreased systemic inflammation, and partially restored gut dysbiosis in a placebo-controlled experiment. XCHT prevented tumour development and increased survival in a mouse model of colorectal cancer under chronic stress. Gut microbiota modification revealed that XCHT’s tumour inhibition was mediated through the TLR4/MyD88/NF-κB pathway, which is why the antitumour effects were connected to gut microbiota. These results emphasise the function of gut microbiota and show the potential of XCHT as an antidepressive and antitumour treatment [55]. Another study examined the effectiveness of probiotics in alleviating complications associated with thyroid hormone withdrawal (THW) in thyroid cancer patients. Fifty patients were randomised to receive either probiotics or a placebo during THW. Probiotics significantly improved symptoms such as lack of energy, constipation, weight gain, and dry mouth while reducing faecal/serum LPS and plasma lipid levels (e.g., cholesterol, triglycerides). THW reduced oral and gut microbial diversity, but probiotics restored it by increasing beneficial gut bacteria such as *Holdemanella*, *Enterococcus*, and *Coprococcus*, while lowering harmful ones like *Fusobacterium*, *Eubacterium ruminantium*, *Ruminococcus*, and *Parasutterella*. In the oral microbiota, probiotics decreased *Prevotella*, *Haemophilus*, *Fusobacterium*, and *Lautropia*, which were linked to dry mouth. These results suggest that probiotics can mitigate THW-related complications by modulating the oral–gut microbiota [56]. A pilot RCT compared the effects of oral preparation and rectal enema on the gut microbiome and postoperative complications in colorectal cancer surgery patients. Forty patients were randomised to receive an oral preparation or rectal enema before surgery. Both groups showed similar changes in gut microbiome composition, with no significant difference in β-diversity on postoperative day 6. Postoperative infections occurred in 32% of patients, regardless of the preparation method. Patients with infections had increased levels of *Actinomycetaceae*, *Actinomyces*, *Sutterella* uncultured species, and *Enterococcus faecalis*. The study suggests that both preparation methods result in similar microbiome disruption, with certain bacteria linked to infections [57]. Another study examined the fungal microbiota in biopsy samples from adenomas and adjacent tissues in the human gut. The dominant fungal phyla identified were Ascomycota, Glomeromycota, and Basidiomycota. The opportunistic pathogens *Phoma* and *Candida* comprised about 45% of the fungal microbiota. Adenomas showed reduced fungal diversity at the operational taxonomic unit (OTU) level, with three OTUs significantly differing from adjacent tissues. Principal Component Analysis (PCA) revealed distinct fungal clusters in advanced and non-advanced adenomas, with four OTUs differing significantly. The adenoma size and disease stage were closely linked to fungal microbiota changes, suggesting potential diagnostic biomarkers for adenomas [58]. Baima et al. (2024) studied periodontitis, oral bacteria, and cancer initiation and progression, highlighting the increased risk of head and neck cancer. The periodontal disease promotes dysbiosis, chronic inflammation, immune evasion, and the spread of carcinogenic bacteria like *Porphyromonas gingivalis* and *Fusobacterium nucleatum*, potentially contributing to malignancies in distant organs such as the digestive tract [59]. Barot studied the tumour microbial profiles of young-onset colorectal cancer (yoCRC) and highlighted potential microbial candidates, such as *Akkermansia* and *Fusobacterium*, to serve as targets for the prevention, diagnosis, and treatment of yoCRC [60]. GI microbiota dysbiosis also contributes to polycystic ovarian syndrome, endometriosis, and cancers, emphasising mechanisms like immune system modulation, the gut–oestrogen axis, and metabolite pathways, highlighting the potential treatments like probiotics, prebiotics, and faecal microbiota transplantation [61].

## 5. Diagnostic and Therapeutic Implications of Gut Microbiota

Kværner et al., 2021 [62] initiated a prospective biomarker CRCbiome study to enhance colorectal cancer screening by developing a non-invasive, microbiome-based classification algorithm to detect advanced colorectal lesions in individuals testing positive for FIT. By analysing the gut microbiome and its interactions with diet, lifestyle, and host factors, the study provided insights into colorectal carcinogenesis. It improved screening sensitivity and specificity, leading to better early detection tools and a deeper understanding of microbiome changes following lesion removal, contributing to CRC prevention and improved public health outcomes [62]. Wisse et al., 2022 [63] aimed to validate the multitarget faecal immunochemical test (mtFIT) as a more sensitive alternative to the FIT for detecting advanced neoplasia (AN) in colorectal cancer screening. A retrospective study indicated that mtFIT showed a higher sensitivity to advanced adenomas and AN than FIT. Prospectively evaluating mtFIT within the Dutch national CRC screening program, this study assessed its clinical utility and potential cost-effectiveness, informing future screening strategies for improved CRC detection [63]. In a randomised study comparing once-only sigmoidoscopy to repeated biennial faecal immunochemical testing (FIT) screening, FIT had higher participation rates and detected more cases of colorectal cancer and advanced adenomas after three rounds compared with sigmoidoscopy. The detection rates for CRC and advanced adenomas increased with repeated FIT screenings, while adverse events were similar between both screening methods. These results suggest that FIT may be more effective for CRC detection over time due to higher participation and cumulative detection [64]. FMT shows promise as a therapeutic approach for colorectal cancer (CRC) by modulating the gut microbiome and enhancing immunotherapy responsiveness [65].

**Figure 3:**
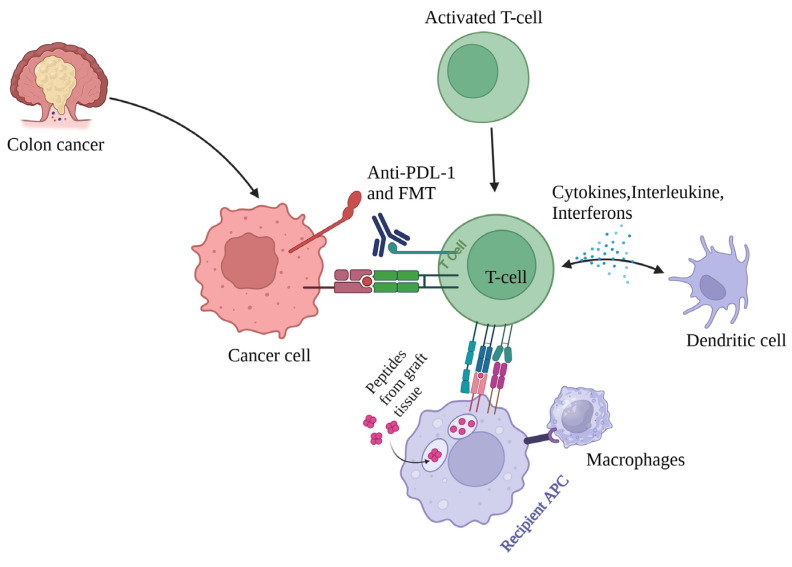
Immune response to FMT and anti-PDL-1 treatment: Faecal microbiota transplantation (FMT) combined with anti-PD-1 immunotherapy changes immune infiltrates, activating CD8^+^ T cells and reducing interleukin-8 myeloid cells, release of cytokines, interleukins and interferons, activating dendritic cells, and increasing the overall efficacy of therapy.

A phase 1 trial of FMT and anti-PD-1 immunotherapy in metastatic melanoma showed clinical responses in 3 of 10 patients, with changes in immune cell infiltrates and gene expression in the tumour microenvironment, suggesting FMT modulates the gut microbiome to influence cancer treatment [66]. FMT combined with anti-PD-1 therapy in 15 patients led to clinical benefits in 6 patients, increasing gut microbiota diversity and CD8^+^ T cell activation, and decreasing interleukin-8 myeloid cells, demonstrating FMT’s potential to overcome anti-PD-1 resistance (Figure 3) [67]. A randomised trial comparing meat-based and pesco-vegetarian diets will test their effects on colorectal cancer risk biomarkers and the gut microbiome. Results clarified that how dietary patterns influence CRC risk through gut–microbiota interactions [68]. In a study on haematologic malignancies, gut microbiome composition varied during CAR-T therapy and correlated with therapeutic response. Patients with severe cytokine release syndrome had distinct microbiome alterations, suggesting the microbiome may affect CAR-T outcomes [69]. Probiotic supplementation reduced the severity of oral mucositis (OM) in nasopharyngeal cancer patients undergoing chemoradiotherapy. The probiotic group showed lower OM rates, enhanced immune response, and improved gut microbiota, indicating probiotics as a protective intervention [70]. Thus, several studies highlight the role of the gut microbiome in cancer treatment, showing that FMT and anti-PD-1 therapy improved responses in melanoma, dietary patterns influence colorectal cancer risk biomarkers, microbiome composition impacts CAR-T therapy outcomes, and probiotics reduce oral mucositis in nasopharyngeal cancer patients undergoing chemoradiotherapy.

## 6. Discussion

The challenges in GI cancer treatment include its high heterogeneity; the desmoplastic environment, which limits drug delivery and supports the growth of the tumour mass; metabolic reprogramming; and the dense stromal microenvironment [71]. The complex relationship between gut dysbiosis and the initiation of various GI and colorectal cancers underscores the crucial roles of inflammation, genotoxicity, and metabolic changes in neoplasia and metastasis [72]. Acute inflammation can stimulate DCs and antitumour immunity or whereas, chronic inflammation can create an environment that favours tumour development and treatment resistance [73]. Developing focused treatment approaches requires understanding the intricate signalling pathways, including NF-kB, JAK-STAT, and MAPK, that mediate inflammatory response. The genotoxicity associated with bacterial toxins such as colibactin and CDT plays a crucial role in the initiation and progression of cancer. The toxins have the ability to damage DNA directly, leading to mutations, chromosomal aberrations, and genomic instability. Colibactin, produced by certain strains of *E. coli*, induces DNA cross-linking and strand breaks, which can result in mutations and disrupt normal cellular processes, promoting tumorigenesis. Similarly, CDT, secreted by bacteria like *C. jejuni* and *H. hepaticus*, causes DNA damage through the induction of double-strand breaks, triggering a cellular response that can lead to cell cycle arrest, apoptosis, or uncontrolled proliferation. Over time, repeated DNA damage and failure to repair the genetic material properly can accumulate, fostering an environment conducive to cancer development, particularly in tissues exposed to these bacterial strains [74]. A method by which bacteria can affect tumour development and metastasis is shown by the interaction between these toxins and host cellular systems; this suggests that more research into microbial virulence factors and their modes of action is necessary. SCFAs and bile acids, metabolic byproducts of gut dysbiosis, impact carcinogenesis [75]. Certain bile acids are linked to neoplastic alterations and DNA damage, while SCFAs have anti-inflammatory qualities and regulate immunological responses. The conflicting roles of microbial metabolites in cancer progression underscores the importance of further research into the role of microbial metabolism in maintaining intestinal balance and its impact on cancer treatment. Since dietary fibres contribute to the production of SCFAs, which support colon health and may reduce the risk of colorectal cancer, it is crucial to explore the relationship between dietary habits and gut microbiota composition [76].

## 7. Future Directions

Research should focus on developing therapeutics that can improve the gut microbiome, including probiotics, prebiotics, and synbiotics, to improve the overall immune response to tumours and restore a healthy balance of gut microbiota. The focus should be on clinical studies to see how well these treatments work alongside traditional cancer therapies. Understanding how gut microorganisms influence cancer development may play a significant role in cancer therapy. This includes studying how they cause genetic damage, produce metabolic byproducts, and regulate the immune system. Knowing more about specific microbial strains and the substances they produce that can prevent or promote cancer will help develop targeted therapies. It is important to conduct long-term studies to explore how dietary habits impact gut microbiota composition and the indicators of colorectal cancer risk. To provide public health recommendations for preventing colorectal cancer, these studies should examine how different diets, especially plant-based versus meat-based, may influence the diversity and function of the gut microbiota. Analysing the gut microbiome could enhance personalised medicine by integrating it into cancer screening and treatment plans. Developing non-invasive biomarkers based on the microbiome to monitor treatment responses and detect advanced colorectal lesions early could significantly improve patient outcomes. Understanding how the gut microbiota influences the body’s response to immunotherapy, including anti-PD-1 medications and FMT, will be essential to enhancing treatment effectiveness. Research goals should focus on elucidating the mechanisms behind microbiome-induced immune changes and identifying the microbial compositions associated with positive treatment outcomes.

## 8. Conclusions

In conclusion, dysbiosis influences colorectal cancer progression in a complex way, and the interplay of factors necessitates a complicated approach to understanding its implications in cancer biology and treatment. By focusing on these future directions, researchers can uncover new strategies to prevent and treat cancer, ultimately improving patient outcomes.

## Figures and Tables

**Table 1:** List of altered bacteria in GI cancers.

S No.	Microorganism	Cancer	Reference
1	*Clostridioides difficile*	Colorectal cancer	[18]
2	*Eubacterium rectale*	Colorectal cancer	[18]
3	*Ruminococcus gnavus*	Colorectal cancer	[18]
4	*Methanobrevibacter smithii*	Colorectal cancer	[18]
5	*Haloferax alexandrinus*	GI cancer	[18]
6	*Helicobacter pylori*	Gastric adenocarcinoma and gastric MALT lymphoma (mucosa-associated lymphoid tissue lymphoma), oesophageal cancer, pancreatic cancer	[19]
7	*Lactobacillus reuteri*	Colorectal cancer	[19]
8	*Neisseria gonorrhoeae*	Prostate and bladder cancer	[19]
9	*Bifidobacterium*	Intestinal, bladder and lung cancer	[20]
10	*Faecalibacterium prausnitzii*	Colorectal cancer and thyroid cancer	[20]
11	*Streptococcus bovis*	Colon cancer	[19,20]
12	*Bacteroides uniformis*	Colorectal cancer	[19,20]

## Abbreviations

AhR	Aryl hydrocarbon receptor
CD	Cluster of differentiation
CRC	Colorectal cancer
GI	Gastrointestinal
IECs	Intestinal epithelial cells
ILC3s	Type 3 innate lymphoid cells
ILs	Interleukins
OM	Oral mucositis
PXR	Pregnane X receptor
SCFAs	Short-chain fatty acids
TNF-α	Tumour necrosis factor-alpha
